# Novel Molecular Insights into Human Lipid-Mediated T Cell Immunity

**DOI:** 10.3390/ijms22052617

**Published:** 2021-03-05

**Authors:** Adam Shahine, Marcin Wegrecki, Jérôme Le Nours

**Affiliations:** 1 Infection and Immunity Program, Department of Biochemistry and Molecular Biology, Biomedicine Discovery Institute, Monash University, Clayton, VIC 3800, Australia; adam.shahine@monash.edu (A.S.); marcin.wegrecki@monash.edu (M.W.); 2 Australian Research Council Centre of Excellence in Advanced Molecular Imaging, Monash University, Clayton, VIC 3800, Australia

**Keywords:** immunity, CD1 molecules, lipids, NKT cells, αβ and γδ T cells

## Abstract

T cells represent a critical arm of our immune defense against pathogens. Over the past two decades, considerable inroads have been made in understanding the fundamental principles underpinning the molecular presentation of peptide-based antigens by the Major Histocompatibility Complex molecules (MHC-I and II), and their molecular recognition by specialized subsets of T cells. However, some T cells can recognize lipid-based antigens presented by MHC-I-like molecules that belong to the Cluster of Differentiation 1 (CD1) family. Here, we will review the advances that have been made in the last five years to understand the molecular mechanisms orchestrating the presentation of novel endogenous and exogenous lipid-based antigens by the CD1 glycoproteins and their recognition by specific populations of CD1-reactive T cells.

## 1. Introduction

T cells represent a key cellular component of defence mechanisms against pathogens and they are subdivided into two main classes based on the genes that encode their T cell receptors (TCRs), namely αβ and γδTCRs. Over the past two decades, most studies on human T cell mediated immunity have focused on the widely studied mechanisms for peptide-based antigens (Ags) displayed by the Major Histocompatibility Complex (MHC) proteins and their subsequent recognition by αβTCRs [1]. However, there exists an additional, evolutionarily conserved family of antigen-presenting molecules, termed the Cluster of Differentiation 1 (CD1), which are broadly expressed on the surface of antigen presenting cells (APCs), including dendritic cells and B cells. CD1 molecules bind and display self- and foreign-lipid-based Ags by sequestering the apolar tails within a hydrophobic groove, while the polar head groups are exposed at the CD1 interface for recognition by specific subsets of T cells [2]. The human CD1 locus encodes four distinct Ag-presenting MHC-I-like molecules, namely CD1a, CD1b, CD1c (group 1), and CD1d (group 2), and structural studies have shown that the size and architecture of each CD1 Ag-binding cleft are markedly different [3], which strongly implies fundamentally different modes of antigen display and lipids repertoire.

## 2. CD1d Recognition by Conventional Natural Killer T Cells

**Figure 1:**
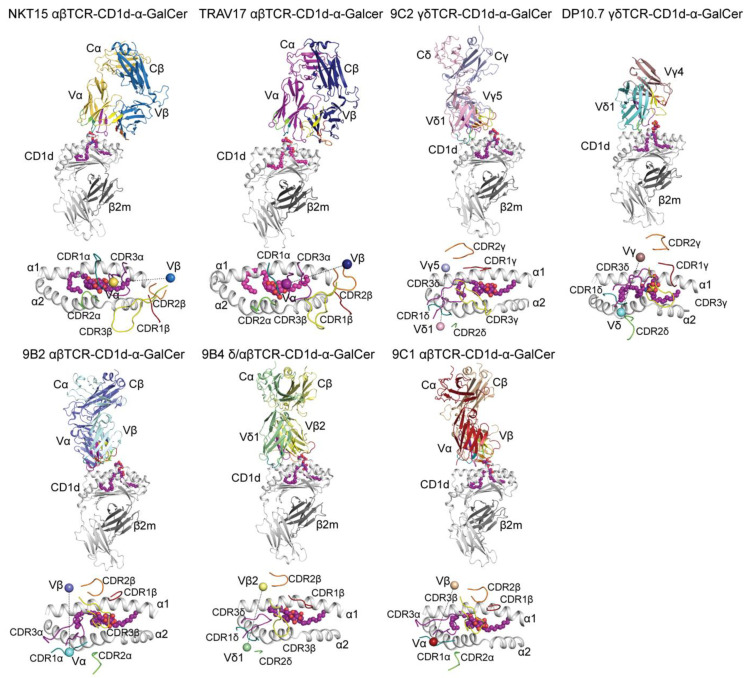
Molecular recognition of CD1d-lipid by human TCRs. Upper panels: Overall cartoon representation of the crystal structure of the NKT15 αβTCR-CD1d-α-GalCer (PDB ID: 1PO6) [8], TRAV17 αβTCR-CD1d-α-GalCer (PDB ID: 4EN3) [14], 9C2 γδTCR-CD1d-α-GalCer (PDB ID: 4LHU) [16], DP10.7 γδTCR-CD1d-sulfatide (PDB ID: 4MNG) [17], 9B4 δ/αβTCR-CD1d-α-GalCer (PDB ID: 4WO4) [18], 9C1 αβTCR-CD1d-α-GalCer (PDB ID: 4WW2) [15], and 9B2 αβTCR-CD1d-α-GalCer (PDB ID: 4WWK) [15] ternary complexes. The variable (V) and constant (C) domains for each TCR chain are labelled. Lower panels: Top-down view of the various TCRs docking atop CD1d (grey). CDR1α (teal), CDR2α (green), CDR3α (purple), CDR1β (red), CDR2β (orange), and CDR3β (yellow) are shown. The center of mass of the variable domains of the TCRs are represented as spheres. Lipid-based antigens are represented as spheres.

Presently, the greatest progress in the field has been centered on CD1d and its recognition by a specialized T cell lineage, namely, Natural Killer T (NKT) cells. There exist two major classes of NKT cells, termed type I and II, with each possessing different functional roles that are directly related to their use of specific TCRs to recognize distinct Ags presented by CD1d [4]. The type I NKT cells are defined by the expression of a semi-invariant αβTCR, comprising an invariant TCR α-chain using the TRAV10-TRAJ18 (in humans) and TRAV11-TRAJ18 (in mice) genes that are associated with a limited array of TCR β-chains (TRBV25-1 in humans; TRBV1, TRBV13, and TRBV29 in mice) [5,6], and by their ability to be potently activated by the archetypal anti-tumor glycolipid, α-Galactosylceramide (α-GalCer). In contrast, type II NKT cells express a more diverse repertoire of TCRs that do not recognize α-GalCer, thus manifesting different ligand specificities when compared to type I NKT cells. Both cell types are essential in protecting us against infections and cancer, yet NKT cells have also the ability to suppress autoimmune diseases [7]. The structural basis underpinning human type I iNKT αβTCR-CD1d-lipid interactions were first illustrated in 2007, whereupon the iNKT αβTCR (NKT15) typically sat in a parallel fashion atop the F’-pocket of CD1d, and directly contacted the carbohydrate headgroup of α-GalCer bound to CD1d via the CDR1α and CDR3α loops (Figure 1) [8]. More recently, the ternary crystal structure of the mouse XV19 type II NKT αβTCR in complex with CD1d presenting the sulfatide/lysosulfatide self- lipid-based antigens [9,10] provided the first molecular insights into the type II NKT αβTCR recognition of CD1d-lipid. Here, in both of the structures, the type II NKT αβTCR docked orthogonally over the extreme end of the A’-pocket of the CD1d binding-cleft. α- and β-chains of the XV19 αβTCR both contributed equally to mediate the interactions at the TCR/CD1d-lipid molecular interface and the CDR3α and CDR3β loops dominated the overall interactions network.

Collectively, NKT cells represent a potentially significant immunotherapeutic target with widespread clinical potential. Accordingly, it has been important to understand the fundamental principles underscoring the development and function of NKT cells and determine how they recognize different lipid-based Ags to initiate an immune response. Here, we will review the most recent advances in our understanding of CD1d-lipids recognition by ‘atypical’ subsets of NKT cells.

## 3. ‘Atypical’ Molecular Recognition of CD1d-Lipid by Human NKT TCRs

### 3.1. CD1d-α-GalCer Recognition by Human ‘Atypical’ NKT αβTCRs

In addition to type I and type II NKT cell subsets, minor populations of human NKT cells that can express a more diverse repertoire of αβTCRs with the ability to bind CD1d-α-GalCer were first identified in human peripheral blood (PBMC) almost two decades ago [11,12,13]. These ‘atypical’ TCRs displayed varied antigen specificities as compared to the canonical iNKT TCRs and, in 2012, Lopez-Sagaseta et al. provided the first insights into the molecular recognition of CD1d-α-GalCer by a TRAV10^−^ NKT αβTCR by determining the crystal structure of the TRAV17-TRAJ18-TRBV25-1 αβTCR-CD1d-α-GalCer ternary complex (Table 1) [14]. Here, although the TCR utilized an α-chain distinct from the invariant TRAV10^+^ iNKT αβTCR, the TRAV17^+^ NKT αβTCR shared a highly conserved CDR3α and it adopted the classical type I iNKT TCR parallel mode of docking over the F’-pocket of CD1d (Figure 1). The CDR1α and CDR3α loops also contributed predominantly to the molecular interactions with α-GalCer whereby the CDR1α made critical contacts with the 4′-OH of α-GalCer. This specific interaction explained the observed lack of reactivity of the TRAV17 αβTCR towards the glycolipid α-GlcCer (α-Glucosylceramide) that differs from α-GalCer by the equatorial positioning of the 4′-OH of the glucose head group versus its axial position in α-GalCer. Despite a conserved overall mode of docking to recognize CD1-lipids, this observation highlighted the fine specificity exhibited by this ‘atypical’ αβTCR against defined lipids relative to the type I NKT αβTCR.

More recently, the identification of additional distinct CD1d-α-GalCer restricted αβ T cell subsets in human PBMC has led to further structural investigations into the molecular basis that underpinned the recognition of lipid-based antigens by TRAV10^−^ NKT αβTCRs [15]. Here, the crystal structures of the 9B2 αβTCR-hCD1d-α-GalCer and 9C1 αβTCR-hCD1d-α-GalCer ternary complexes (Table 1) revealed that these ‘atypical’ NKT αβTCRs adopted distinct orthogonal docking strategies in recognizing α-GalCer, whereby the 9B2 and 9C1 αβTCRs sat atop the A’-pocket of the CD1d antigen-binding groove with docking angles of ~110° and ~75°, respectively (Figure 1) [15]. These docking topologies differed drastically from the parallel mode of docking that was employed by type I iNKT αβTCRs and were more reminiscent of the docking strategy adopted by the mouse type II NKT αβTCRs [9,10]. Interestingly, as opposed to the type I iNKT αβTCR, which exclusively utilized the CDR1α and CDR3α to interact with α-GalCer, the CDR1α, CDR2β and CDR3β loops were all involved in recognizing α-GalCer in the 9C1 αβTCR ternary complex while the 9B2 αβTCR solely relied on the CDR3β loop to recognize the glycolipid (Figure 1). These alternative recognition strategies that were exhibited by these newly identified ‘atypical’ CD1d-α-GalCer-restricted αβTCRs clearly underpinned the varied molecular interactions that were involved between the αβTCRs, CD1, and the lipid-based Ag.

### 3.2. Beyond the Molecular Recognition of CD1d-Lipids by αβTCRs

In addition to the characterization of TRAV10^−^ αβTCRs that exhibited varied reactivities to CD1d-α-GalCer and differing docking modes compared to the type I NKT αβTCR, additional studies demonstrated that the CD1d-lipids reactive T cell repertoire in human PBMCs was not solely restricted to αβ T cells, and also included γδ and δ-αβ T cells [16,17,18,19]. In 2012, Bai et al. identified human CD1d-sulfatide restricted γδ T cells that were predominantly Vδ1^+^ and exhibited highly diverse CDR3 sequences among various donors [19]. Similarly, the gene usage of the characterized human CD1d-α-GalCer restricted γδTCRs was also heavily biased towards Vδ1, while the Vγ-chain sequences were more diverse [16]. The first two crystal structures of a γδTCR-CD1d-lipid ternary complex provided the first molecular insights into the recognition of CD1-lipids by γδTCRs (Figure 1) (Table 1) [16,17]. Here, human DP10.7 and 9C2 γδTCRs both sat atop the CD1d antigen-binding cleft, whereby the 9C2 γδTCR adopted a more A’-pocket centric docking strategy that was in clear contrast to the classical parallel mode of docking over the F’-pocket by the type I iNKT αβTCR and more reminiscent of a type II NKT binding mode [9,10] (Figure 1). The δ1-chain of both γδTCRs played a predominant role in interacting with the CD1d molecule, notably via two consecutive and conserved Trp residues within the CDR1δ loop. However, whilst the CDR3δ loop of the DP10.7 γδTCR was the key structural determinant to recognize the sulfatide antigen, the 9C2 γδTCR engaged α-GalCer via the CDR3γ loop (Figure 1). Additionally, a novel population of CD1d-α-GalCer reactive T cells termed δ/αβ T cells was identified in the PBMC of healthy humans. δ/αβ T cells represented <1% of the total CD1d-α-GalCer reactive T cells in most donors, yet this frequency reached 50% in one individual. Here, similarly to the ‘atypical’ 9C1, 9B2 αβTCRs, and the 9C2 γδTCR [15,16], the 9B4 δ/αβTCR adopted a clearly distinct mode of binding of the classical type I iNKT TCR, whereby the δ/αβTCR docked orthogonally over the A’-pocket of CD1d.

Collectively, these recent studies clearly demonstrated that NKT cells carry a range of TCRα, TCRβ, TCRγ, and TCRδ chains that can impact on α-GalCer specificity and provide a greater scope for diverse glycolipid antigens recognition by ‘atypical’ CD1-restricted T cell subsets. Further, these five recent crystal structures [15,16,17,18] highlighted the versatility of CD1d-lipid recognition by TCR and, ultimately, expanded our understanding of T cell biology and radically reshaped our understanding of NKT TCR recognition.

## 4. Molecular Presentation of Novel Self-Lipids by Group 1 CD1

### 4.1. Presentation of Self-Lipids by CD1a in Human Skin

Certain features of human CD1a make it unique within the CD1 family. Although it can be found on thymocytes, innate lymphoid cells, and some dendritic cells (DCs) in the periphery, CD1a is highly expressed on Langerhans cells in the epidermis [20]. It mostly localizes on the cell surface because it lacks the cytosolic tyrosine-based sorting motif and does not undergo extensive intracellular processing [21]. CD1a becomes readily available to capture a broad range of foreign lipid-based antigens that come into contact with human skin due to its constitutive presence at an important immunological barrier site [22]. Furthermore, it has been shown that there exists a large number of endogenous molecules that CD1a can capture and present to autoreactive T cells [23,24,25].

**Figure 2:**
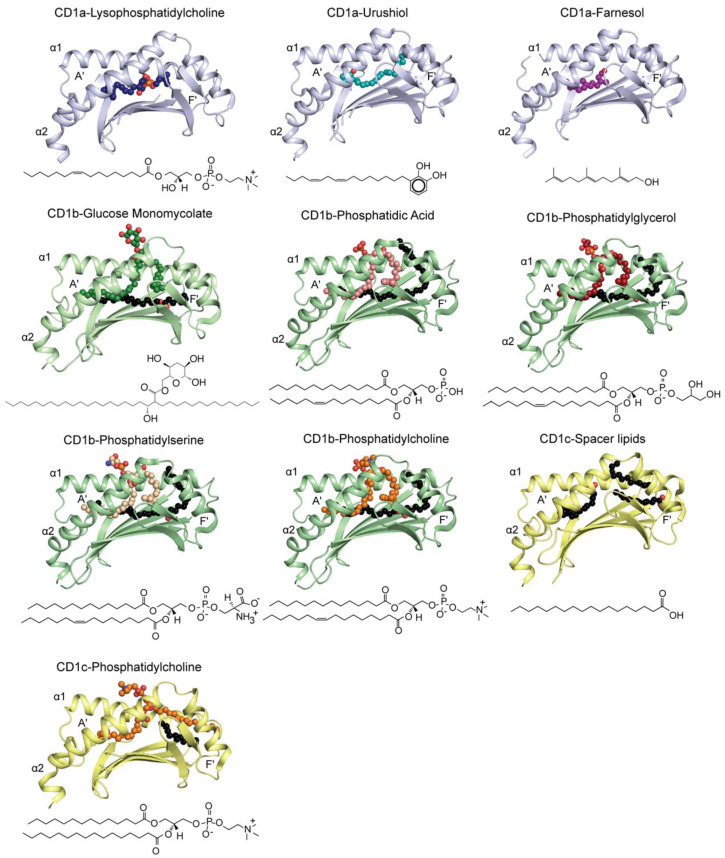
Crystal structure of the most recent group 1 CD1-lipid antigens binary complexes. CD1a (blue) presenting lysophosphatidylcholine (LPC) (PDB ID: 4X6E) [25], urushiol (PDB ID: 5J1A) [28], farnesol (PDB ID: 6NUX) [29]; CD1b (green) presenting glucose monomycolate (GMM) (PDB ID: 5L2J) [32], phosphatidic acid (PA) (PDB ID: 5WKG) [33], phosphatidylglycerol (PG) (PDB ID: 5WL1) [33], phosphatidylserine (PS) (PDB ID: 5WKE) [33], phosphatidylcholine (PC) (PDB ID: 6D64) [34]; CD1c (yellow) presenting spacer lipids (PDB ID: 5C9J) [31], and phosphatidylcholine (PDB ID: 6C15) [35]. The CD1 antigen-binding clefts and presented lipids are represented as cartoon and spheres, respectively. The chemical structures of the presented lipids are also shown.

The crystal structure determination of a number of CD1a-lipid binary complexes demonstrated that the antigen-binding cleft of CD1a is relatively small (~1600–1800 Å^3^) relative to other CD1 isoforms [25,26,27,28,29] (Table 2) (Figure 2). It can be divided into a narrow enclosed and extremely hydrophobic curved tunnel, named A’-pocket, and a bulkier solvent exposed F’-pocket. While the A’-pocket is covered by a rigid platform termed A’-roof, the antigenic compounds gain access to the cavity of the protein through the F’-portal, a ~10 Å wide opening above the F’-pocket. Although a similar organization of the binding cleft can be observed in all of the CD1 proteins [30,31], the CD1a portal is not positioned in the center of the α1/α2 domain, but it is clearly shifted towards one side of the groove. This particular arrangement results in a large continuous surface atop CD1a that remains mostly unaffected by the nature of the presented lipid. This has important structural consequences for mechanisms of lipid presentation and recognition.

While initial crystallographic studies clearly demonstrated that the A’-pocket of CD1a can easily accommodate one acyl chain, the remaining part of the ligand can adopt a variable position within the F’-pocket and, depending on its size and polarity, it can be partially or fully solvent exposed through the F’-portal [25,26,27,28,29]. Accordingly, in the sphingomyelin-bound CD1a binary structure, the 24-carbon long acyl tail spans across the whole binding cleft and anchors deeply in the most distal segment of the A’-pocket. At the same time, the shorter sphingosine unit runs in parallel to the bottom of the cleft towards the A’/F’ junction. Subsequently, the phosphocholine headgroup protrudes through the F’-portal and it impacts on the edge of the A’-roof, where a network of electrostatic interactions is disrupted. It has been postulated that the altered architecture of the topmost part of CD1a adversely impacts the recognition by autoreactive T cells. In contrast, the same study showed that smaller lipids, such as lysophosphatidylcholine (LPC), could be fully accommodated in the cleft of CD1a and it did not significantly alter the overall architecture of the protein. Here, the A’-pocket is again filled with the acyl tail; nevertheless, the lack of a second chain and a reduced length of the lipid anchor (18C) result in only a minimally solvent exposed phosphocholine headgroup (Figure 2). In this setting, the A’-roof of CD1a remains intact, and it can be potentially recognized by T cells. Further functional and structural studies revealed that many other compounds commonly found in human skin, such as components of sebum, can elicit a CD1a-mediated immune response [23]. It is yet to be elucidated how exactly CD1a presents headless skin oils that include squalene, wax esters, and diacylglycerides. The most recent models suggest that those compounds will remain buried within the A’/F’ pockets of CD1a in a similar fashion to LPC [36]. Interestingly, an analogous scenario has been suggested for certain small apolar molecules of non-pathogenic origin, such as urushiol, found in poison ivy, and farnesol, a compound that is routinely used in cosmetics [28,29]. Both of the molecules are believed to activate CD1a-restricted T cells by a lack of interference (Figure 2).

### 4.2. CD1b Presenting Self- and Microbial-Lipids

Of the members of the group 1 CD1 family, the most is known about the molecular mechanisms of lipid antigen presentation by CD1b and it can be categorized based on the class of lipid-based antigens: being foreign lipids from *Mycobacterium tuberculosis*, and either self- phospholipids or sphingolipids. Recent advances in the field have resulted in significant structural characterization of phospholipid presentation by CD1b, with the structures of CD1b-phosphatidic acid (PA), phosphatidylserine (PS), phosphatidylglycerol (PG), and phosphatidylcholine (PC) complexes being determined (Table 2) [33,34]. Despite differences in acyl tail lengths, the sn2 and sn1 acyl tails of each phospholipid are bound within the A’- and C’-binding pockets of CD1b, respectively, whereby the C’-pocket is a unique feature to CD1b, and it loops underneath the α2-helix hinge region (Figure 2). Furthermore, each acyl tail overlays tightly within the binding groove, forcing solvent exposure of the polar phosphate headgroup for TCR recognition.

CD1b can occupy lipids of C60 to C80 carbons in length due to the unique size of its antigen binding pocket [2]. The mechanism of presenting these large lipid antigens is demonstrated best in CD1b presentation of C60 glucose-monomycolate (GMM). Here, the β-hydroxy fatty acid occupies the A’, T’, and F’-pockets, with the α-alkyl side chain bound within the C’-pocket [37]. Again, the polar glucose headgroup of GMM is solvent exposed, allowing for direct recognition by CD1b-GMM specific TCRs [32,38]. In contrast, self-lipid presentation of phospholipids and sphingolipids are limited to the A’- and C’-pockets, as there are no naturally existing cellular lipids that exhibit the same tail lengths as GMM. As such, CD1b is ideally suited to the presentation of mycobacterial mycolates for T cell mediated immune activation via evolutionary conserved mechanisms. Because self-lipids do not fill the entirety of the CD1b antigen binding pocket, a secondary cellular scaffold lipid(s) is bound within the T’- and F’-pockets. The exact species and function of these scaffold lipids are presently unknown; however, previous investigations have identified highly hydrophobic and headless candidates, such as wax esters or 1,3-diacylglycerols [39]. These scaffold lipids are believed to retain structural integrity of the CD1b antigen binding pocket in the absence of a single lipid species that occupies the entirety of the pocket. These scaffold lipids are observed in each CD1b-phospholipid structure determined, with the electron density for each distinctly separate from the presented phospholipid [30]. Because these observations are consistent across all CD1b-self-lipid crystal structures determined to date, this has given rise to a ‘two-compartment’ model of lipid presentation: whereas, in the absence of a single mycolate lipid that occupies the entirety of the CD1b antigen pocket, the presented self-lipid occupies the A’/C’-pockets, and the scaffold lipid occupies the T’/F’-pockets [40].

### 4.3. The Molecular Diversity of Lipids Presentation by CD1c

**Figure 3:**
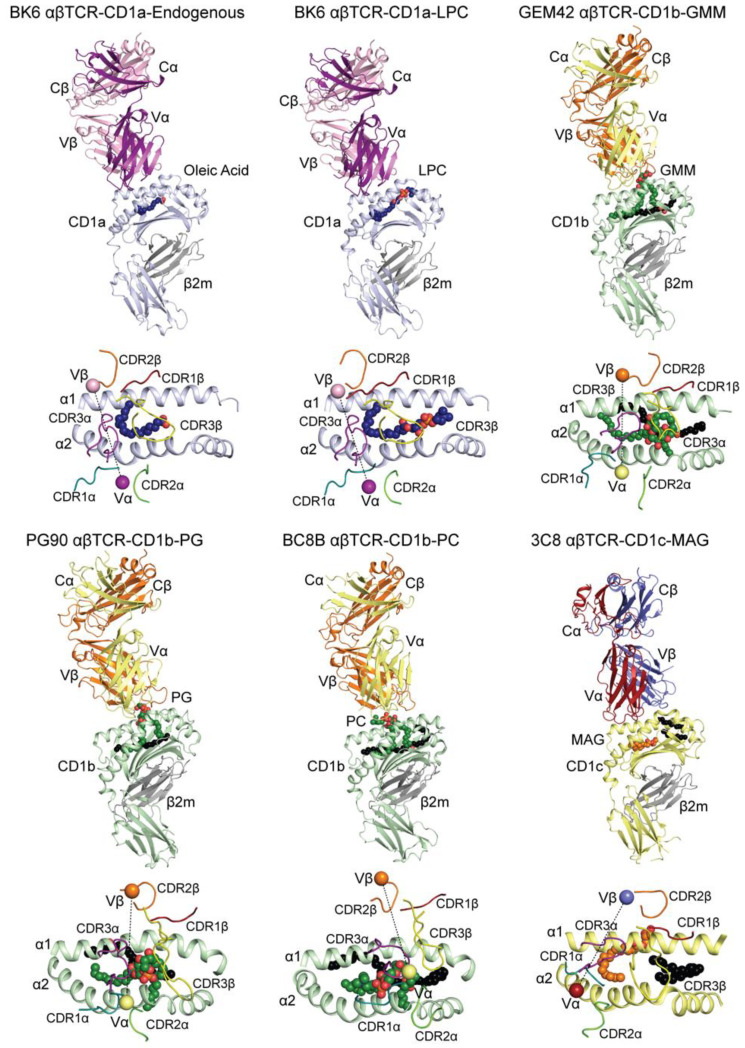
Crystal structures of αβTCRs in complex with group 1 CD1-lipid. Upper panels: Cartoon representation of the crystal structure of the BK6 αβTCR-CD1a-endo (PDB ID: 4X6D) [25], BK6 αβTCR-CD1a-lysophosphatidylcholine (LPC) (PDB ID: 4X6C) [25], GEM42 αβTCR-CD1b-glucose monomycolate (GMM) (PDB ID: 5L2K) [32], PG90 αβTCR-CD1b-phosphatidylglycerol (PG) (PDB ID: 5WKI) [33], BC8B αβTCR-CD1b-phosphatidylcholine (PC) (PDB ID: 6CUG) [34], and 3C8 αβTCR-CD1c-monoacyl glycerol (MAG) (PDB ID: 6C09) [35] ternary complexes. CD1a (blue), CD1b (green), CD1c (yellow), and β2m (grey). The lipids are shown as colored spheres. Lower panels: top-down view of the TCRs docking atop the CD1 molecules. The CDR loops are colored as per Figure 1, and the center of mass of the variable domains of the TCRs are represented as spheres.

By comparison to CD1a and CD1b, our understanding of CD1c-lipid presentation is significantly more limited. Despite the established importance of CD1c in the presentation of mycobacterial lipid Ags mannosyl-phosphomykoketide (MPM) and phosphomykoketide (PM) [41,42], only recently have the mechanisms of self-lipid presentation been elucidated. The determination of CD1c presenting spacer lipids (CD1c-SL) reveals a single C18 oleic acid in the A’-pocket, and two stacked C12 hydrocarbon lipids in the F’-pocket (Figure 3) [31]. Furthermore, the molecular dynamics of this structure reveal that lipid occupancies within the F’-pocket are a major determinant of CD1c structural viability. Recently, the structure of CD1c presenting endogenous self- lipids was determined. Based on normal phase liquid chromatography-mass spectrometry (LC-MS), phosphatidylcholine, and sphingomyelin, with tail lengths of 34:1, were the prevalent lipid eluents. As such, both C34:1 PC (Figure 3) and sphingomyelin could be modelled into the electron density of the CD1c-endo crystal structure [35]. In both cases, the longer sn2 C18:1 lipid tail was modelled into the A’-pocket, with the phosphocholine headgroup being exposed to the solvent for T cell recognition—a mechanism that was observed with the presentation of phospholipids presented by CD1b (Figure 2). In both structures, clear electron densities for two lipid tails stacked upon each other were visible within the F’-pocket. At the base of the F’-pocket, a 12 carbon-long fatty acid could be modelled, whereas, for CD1c-endo, the sn1 lipid tail could be built above the 12-carbon fatty acid spacer (Figure 2). As such, the upper chamber was dubbed the G’-pocket to separate the two and it is a unique feature of the CD1c antigen binding groove.

## 5. Molecular Mechanism Underpinning the Recognition of Group 1 CD1-Restricted Self-Lipids

### 5.1. Beyond the Left-Right Mismatch Recognition of CD1a

It is evident that CD1a-restricted autoreactive T cells are highly abundant in human peripheral blood and they play an important role in skin autoimmunity [43,44]. However, our current understanding of the molecular mechanisms behind CD1a-mediated autoimmune response is largely based on only two ternary complex structures (Table 2). Birskinshaw et al. showed that the autoreactive BK6 αβTCR (TRAV12-3/TRBV6-2) mostly recognizes the A’-roof of CD1a carrying smaller lipids (LPC and oleic acid), leaving the F’-portal relatively uncovered. Because some of the larger autoantigens, such as sphingomyelin, can interfere with the binding of the BK6 TCR, it was postulated that those molecules that modify the roof architecture can hinder TCR recognition and, thus, act as inhibitory ligands; therefore, only their displacement by smaller lipids, such as LPC, can lead to T cell stimulation. In line with this model, other small apolar compounds, such as aforementioned urushiol and farnesol, can act as permissive antigens inducing CD1a-dependent responses and leading to contact hypersensitivity or allergies [29]. Further, in a recent study, CD1a molecules carrying a heterogeneous array of lipids were assembled into tetramers and then bound a large number of T cells from human skin confirming the major role of the A’-roof of CD1a in T cell recognition independent of the antigen cargo [24]. Moreover, recent functional data on house dust mites (HDM) or bee and wasp venom allergies showed that, often, the frequency of CD1a-restricted autoreactive T cells is much higher in sensitive individuals [45,46,47]. In each case, it is believed that the phospholipase A2, an enzyme that is capable of hydrolyzing endogenous phospholipids, can generate an array of novel CD1a-specific antigens, which, in turn, act as permissive ligands that activate T cells and stimulate the production of INFγ, IL-13, or GM-CSF [47]. Similarly, it has been shown that, in psoriasis, the PLA2-induced antigen production results in an increased CD1a-dependent immune response [48]. Moreover, the role of CD1a and autoreactive T cells in skin inflammation was further confirmed in psoriatic individuals and using CD1a-expressing transgenic mice [28]. It is becoming clear that, under normal circumstances, there is a large population of CD1a-restricted T cells, but their activation tightly depends on an interplay between stimulatory and inhibitory self-lipids. When that equilibrium is altered either by exogenous factors (PLA2, small chemicals) or by an increased frequency of CD1a-restricted T cells (allergic individuals), it can lead to an exaggerated immune response and skin disorders.

### 5.2. Left/Right-Centric Mechanism of CD1b-Lipid Recognition in Diseases

Until now, most of the progress into our understanding of the molecular recognition of group 1 CD1 molecules by TCRs has been made on CD1b, notably via the structural determination of three αβTCR-CD1b-lipid ternary complexes [32,33,34]. These include TCRs that expressed the semi-invariant germline-encoded mycolyl-reactive (GEM) T cells [38,49], which exhibit specific reactivity towards CD1b presenting mycolate-derived lipids, which are key constituents of the *Mycobacterium tuberculosis* cell wall. These lipids may lack a polar headgroup (mycolic acid) or contain glucose (glucose-monomycolate; GMM), or glycerol (glycerol-monomycolate; GroMM) polar moieties. The structural determination of CD1b presenting GMM in complex with the GEM TCR, GEM42 (TRAV1-2/TRBV6-2) reveals the molecular mechanisms of mycobacterial lipid recognition. Here, the CDR3α/β regions form a tweezer-like mechanism, generating an electrostatically positive pocket, which specifically recognizes the glucose moiety of the lipid antigen, allowing for the differentiation between GMM and other mycolate species [32]. This similar mechanism is observed in the recognition of the phospholipid phosphatidylglycerol (PG) by the PG90 αβTCR (TRAV26-1/TRBV7-8), where the CDR3α/β regions recognize the negatively charged phosphoglycerol moiety [33]. Despite the differences in TCR gene usages, T cell function, and lipid reactivities, these TCRs dock onto CD1b via a conserved left-centric mechanism, whereby each TCR docks over the CD1b A’-pocket and directly interacts with the solvent exposed polar lipid headgroup. Whilst the definitive role of the GEM T cells lies in adaptive immune activation towards mycobacterial infection, the role of phospholipid reactive T cells, particularly PG-reactive T cells [50], is vague. In mammalian cells, PG is found in trace amounts that are localized within mitochondria, and it is a precursor molecule in the synthesis of cardiolipin [51,52]. However, PG is highly abundant in bacterial cellular membranes, and it stands to act as a bacterial-lipid antigen upon presentation by CD1b. Despite this, it is yet to be determined whether the role of these CD1b-PG reactive T cells is in the context of bacterial infection or in an autoreactive manner, whereby PG can be released from mitochondria upon oxidative stress [50].

Irrespective of the class of the lipid, the GEM42 and PG90 TCRs interact with CD1b presenting specific lipid antigens in a similar manner. Recently, the mechanisms of recognition of a newly identified T cell subset that, instead, broadly recognizes specific lipid classes have been elucidated. In particular, the BC8B αβTCR (TRAV9-2/TRBV6-2) that was sequenced from a recently isolated T cell line from PBMCs of a healthy blood donor exhibits specific recognition towards a broad range of phospholipid antigens has been structurally characterized in complex with CD1b presenting phosphatidylcholine (PC) (Figure 3) [34]. This interaction is mediated by the CDR1α and CDR3α loops and, while they specifically recognize the phosphate moiety, do not completely encapsulate the presented lipid headgroup. Instead, the CDR1/3α regions form an escape channel over the A’-region of CD1b, which allow the specific phospho-moiety of each phospholipid species to escape [34]. Furthermore, the TCR itself docks over the CD1b F’-portal in a right-centric manner. This docking mechanism differs from the specific mechanism of the PG90 and GEM42 αβTCRs (Figure 3) and, instead, shares closer similarities to the type I NKT TCR recognition of CD1d (Figure 1). While these mechanisms of recognition are novel for CD1b, it does not ascertain to the role of these broad-phospholipid reactive T cell. Recently, autoreactive T cells have been found to contribute towards both antitumor immunity in T cell lymphoma and hyperlipidemia through the recognition of CD1b presentation of self-phospholipids. HJ1 T cells from a double transgenic mouse model expressing group 1 CD1 (hCD1Tg/HJ1Tg) exhibited significant IFN-γ and IL13 secretion in vitro upon activation by CD1b presenting self-phospholipids, including PC and phosphatidylethanolamine (PE), which suggests a further role of phospholipid presentation in autoimmunity [53,54].

While significant headway has been made into understanding CD1b-restricted TCR recognition, presently much remains unknown. For instance, the mechanisms of TRBV4-1 TCR recognition, which include LDN5-like TCRs, are unknown, despite accounting for approximately 40% of known CD1b restricted TCRs, and exhibiting reactivity towards both bacterial- and self- lipid antigens [55]. Preliminary alanine-scanning mutagenesis and structural studies of TRBV4-1 TCRs reveal key amino acid residues in the CDR1β (His29β and Arg30β) and CDR2β (Tyr58β) regions that may directly contact CD1b or influence the positioning of the CDR3β region for antigen recognition [55]. Furthermore, three CD1b-restricted γδTCRs encoding Vδ1 gene usage were identified, each exhibiting one of two distinct modes of recognition towards bacterial glycolipid BBGL2 lipid from *Borrelia burgdorferi* and a range of self-lipids, via ligand dependent or independent mechanisms [56]. Whether or not the γδTCR ligand dependent mode of recognition shares similarities with the GEM42 or PG90 αβTCRs, or whether ligand independent recognition is mediated by interactions away from the F’-portal, similarly to the G7 γδTCR-MR1-5-OP-RU ternary complex in which the γδTCR interactions with MR1 are predominantly dominated by the Vδ1 chain and the α3-domain of MR1 underneath the antigen-binding cleft [57], presently remain unknown.

### 5.3. Beyond the Buried Ligand Model of CD1c Recognition

In comparison to CD1a and CD1b, our understanding of αβTCR recognition of CD1c is presently limited to a unique ternary complex. The 3C8 αβTCR (TRAV29/DV5/TRBV7-2) (Table 2) only interacts with CD1c in the presence of permissive ligands, such as monoacylglycerol (MAG); that is, when presented lipids are completely sequestered internally within the CD1c antigen binding pocket [35]. These buried ligands permit the formation of the A’-roof, as observed with BK6 αβTCR recognition of CD1a, which provides a platform of recognition of CD1c by the 3C8 αβTCR in a ligand-independent manner (Figure 3). While these data provide the first insights into αβTCR recognition of CD1c, the mechanisms of how TCRs can recognize lipid antigens with solvent exposed polar headgroups are yet to be observed. For instance, two TCRs, both exhibiting unique Vα and Vβ, denoted as the double negative DN6 (TRAV36/TRBV6-6) [58] and the CD8^+^ CD8.1 αβTCR (TRAV8-6/TRBV20-1) [59], can differentiate between CD1c presentation of *Mycobacterium tuberculosis* mykoketides PM and MPM, respectively [42]. This suggests a recognition mode that differs from 3C8 buried ligand recognition, and that is more akin to the CD1b-restricted TCR recognition. Furthermore, the CD8^+^ 22.5 αβTCR (TRAV26-2/TRBV7-9) has demonstrated in vitro recognition CD1c presentation of both PM and MPM [42], which may involve a mode of recognition that differs from both the 3C8 buried ligand model and the predicted DN6/CD8.1 ligand dependent models of recognition. In contrast, CD1c-autoreactive T cells have demonstrated a conserved TRBV4-1 gene usage, with a subset of CD4^+^/TRBV4^+^ T cells characterized by CD154 upregulation and cytokine production [60]. Here, mutational mapping of the TRBV4-1 sequence revealed that Arg30β and Tyr51β play a crucial role for CD1c recognition, which shares some similarities to CD1b recognition by CD1b-restricted TCRs [55]. Furthermore, tetramers of CD1c-C32 PM are able to detect a subset of Vδ1^+^ γδ T cells from healthy PBMCs that demonstrate T cell activation. Despite a conservation in their Vδ1 usage, each isolated TCR clone exhibits significant variation in their CDR3 sequences, as well as their γ usages, which modulate ligand affinities [61]. This is clearly observed in the case of the TCR 12.16-3 (TRDV1-TRDD2/3/TRGV3-TRGJP2), which demonstrates in vitro cross reactivity with sulfatide and lysophospholipids. Despite the varied nature of these γδTCRs, the modes of ligand recognition and differentiation, as well as cross reactivity, are yet to be structurally explored.

## 6. Conclusions

There is a growing evidence that lipid-based antigens play an essential role in modulating immune responses within the human body. Here, we discussed the molecular features of CD1 proteins that all contain hydrophobic clefts of variable shape and size that allow them to bind and present a vast array of antigenic molecules. We described our current understanding of how those protein-lipid complexes can be recognized by αβ and γδ T cell receptors via variable docking mechanisms that occasionally do not require direct contact with the lipid itself and, in many cases, differ from the MHC-peptide recognition paradigm. Moreover, we summarized the most recent reports showing that altered function of CD1 proteins and their recognition by T cells can be linked to the pathology of cancer, psoriasis, and many other autoimmune diseases, making the CD1 field an attractive target of clinical research.

## Figures and Tables

**Table 1:** Crystal structures depicting ‘atypical’ T cell receptor (TCR) recognition of CD1d.

T Cell Receptor	T Cell Type	PDB ID	References
TRAV17	αβ	4EN3	[14]
9C1	αβ	4WW2	[15]
9B2	αβ	4WWK	[15]
9C2	γδ	4LHU	[16]
DP10.7	γδ	4MNG	[17]
9B4	δ/αβ	4WO4	[18]

**Table 2:** Group 1 CD1-lipid crystal structures (last five years).

Lipids	Source	Type	PDB ID	References
**CD1a**				
Lysophosphatidylcholine	Self (Commercial)	Ternary	4X6C	[25]
Endogenous fatty acids	Self (expression system)	Ternary	4X6D	[25]
Lysophosphatidylcholine	Self (Commercial)	Binary	4X6E	[25]
Sphingomyelin	Self (Commercial)	Binary	4X6F	[25]
Urushiol	Plant (commercial)	Binary	5JIA	[28]
Farnesol	Balsam of Peru (commercial)	Binary	6NUX	[29]
**CD1b**				
Glucose monomycolate C36	Bacterial (purified)	Binary	5L2J	[32]
Glucose monomycolate C36	Bacterial (purified)	Ternary	5L2K	[32]
Phosphatidylserine	Self (commercial)	Binary	5WKE	[33]
Phosphatidic Acid	Self (commercial)	Binary	5WKG	[33]
Phosphatidylglycerol	Bacterial (commercial)	Ternary	5WKI	[33]
Phosphatidylglycerol	Bacterial (commercial)	Binary	5WL1	[33]
Phosphatidylcholine	Self (commercial)	Ternary	6CUG	[34]
Phosphatidylcholine	Self (commercial)	Binary	6D64	[34]
**CD1c**				
Spacer lipids	Refolding	Binary	5C9J	[31]
Monoacyl glyceride	Self (expression system)	Ternary	6C09	[35]
Phosphatidylcholine	Self (expression system)	Binary	6C15	[35]

## Data Availability

Not applicable.

## Abbreviations

APC	Antigen presenting cell
CD1	Cluster of differentiation 1
CDR	Complementarity determining region
iNKT	Invariant Natural Killer T cells
MHC	Major histocompatibility complex
PBMC	Peripheral blood mononuclear cell
TCR	T cell receptor
TRAV	Human T cell receptor alpha variable gene
TRBV	Human T cell receptor beta variable gene
TRDV	Human T cell receptor delta variable gene
TRGV	Human T cell receptor gamma variable gene
